# The influence of perioperative interventions targeting psychological distress on clinical outcome after total knee arthroplasty

**DOI:** 10.1007/s00296-020-04644-y

**Published:** 2020-07-29

**Authors:** Juliette Caroline Sorel, Geke Marianne Overvliet, Maaike Gerarda Johanna Gademan, Chantal den Haan, Adriaan Honig, Rudolf Wilhelm Poolman

**Affiliations:** 1 Department of Orthopaedics, Leiden University Medical Center, 2333 ZA Leiden, The Netherlands; 2 Department of Orthopaedic Surgery and Traumatology, Joint Research OLVG, 1091 AC Amsterdam, The Netherlands; 3 Department of Psychiatry, OLVG, Amsterdam and Amsterdam UMC, Jan Tooropstraat 164, 1105 AZ Amsterdam, The Netherlands; 4 Department of Clinical Epidemiology, Leiden University Medical Center, 2333 ZA Leiden, The Netherlands; 5 Medical Library, Department of Research and Education, OLVG, 1061 AE Amsterdam, The Netherlands; 6 Department of Orthopaedic Surgery and Traumatology, OLVG, Oosterpark 9, 1090 HM Amsterdam, The Netherlands

**Keywords:** Total knee arthroplasty, Psychological distress, Pain, Function, Quality of life

## Abstract

Our aim was to assess the effect of perioperative interventions targeting psychological distress on clinical outcome after total knee arthroplasty (TKA). We searched studies on the effect of perioperative interventions focused on psychological distress used in conjunction with TKA on pain, function, and quality of life (QoL) on PubMed, Embase.com, PsycINFO/OVID, CENTRAL, the Cochrane Database of Systematic Reviews, Scopus, and Web of Science. We included 40 studies (22 RCTs, ten cohort studies, and eight quasi-experimental studies) with a total of 3846 patients. We graded the quality of evidence as low for pain and function and as moderate for QoL. Patients receiving music, education, cognitive behavioural therapy, guided imagery, pain coping skills training, Reiki, occupational therapy with self-monitoring, and biofeedback-assisted progressive muscles relaxing training had lower pain scores or declined opioid prescriptions after TKA. Pain coping skills training, audio recording-guided imagery scripts, video promoting self-confidence, psychological therapies by video, Reiki, music, occupational therapy with self-monitoring, education, and psychotherapy improved postoperative functional outcome. Education through an app improved QoL after TKA. The studies in our systematic review show that perioperative interventions targeting psychological distress for patients receiving TKA seem to have a positive effect on postoperative pain, function, and QoL. RCTs with strict methodological safeguards are still needed to determine if perioperative interventions focused on psychological distress should be used in conjunction with TKA. These studies should also assess which type of intervention will be most effective in improving patient-reported outcome measures and declining opioid prescriptions.

**Electronic supplementary material:**

The online version of this article (10.1007/s00296-020-04644-y) contains supplementary material, which is available to authorized users.

## Introduction

Total knee arthroplasty (TKA) is the treatment of choice for medically operable patients with end-stage osteoarthritis (OA) of the knee joint if non-surgical therapies fail to obtain adequate pain relief and functional improvement [1]. TKA proved to be a cost-effective procedure with excellent postoperative implant-related outcomes, such as radiographic appearance and implant features [2]. Nevertheless, a significant number of patients report pain (8.0–26.5%) on long-term follow-up after TKA [3] and as many as 11–19% of the patients are not satisfied with their procedure [4, 5]. Persistent pain after TKA is commonly treated with opioids after surgery [6]. Currently, increasing misuse and addiction to opioids are a rapidly evolving public health issue [7]. Improving pain scores after surgery by understanding factors influencing postoperative pain may help prevent further expansion of this opioid crisis [7].

Unfavourable outcome after TKA is related to age, gender, level of education, pre-operative function and pain [8], comorbidities [9], social support [9], Body Mass Index (BMI) [10], and surgical factors [11–13]. Preoperative psychological factors such as mental health status, symptoms of anxiety and depression, and poor coping skills have also been examined [13–15]. Systematic reviews [16–18] and meta-analyses [19, 20] on this subject reported that psychological distress might affect the postoperative outcome (pain and function) after TKA. Perioperative interventions targeting these psychological factors may improve clinical outcome after surgery. Previous studies have examined the effect of interventions influencing psychological factors to improve postoperative clinical outcome after TKA [21–24]. We found three previous systematic reviews on psychological interventions in conjunction to orthopaedic surgeries [25–27]. The systematic review of Bay et al. [25] did not support the effectiveness of psychological interventions in improving patient-reported joint outcomes after TKA as the interventions explored by studies were found to be ineffective at the latest follow-up. The results of Szeverenyi et al. [26] and Tong et al. [27] indicated that psychological interventions might improve postoperative outcome of orthopaedic surgery. These previous reviews included several types of orthopaedic procedures (among which TKA, total hip arthroplasty (THA) and spinal procedures) and did not focus on TKA. Besides, the most up-to-date search was performed in January 2018 [27].

To our knowledge, focused systematic reviews of studies on TKA patients with wide search and inclusion criteria investigating the effect of interventions targeting psychological distress on patient-reported outcome measures pain, function and/or quality of life (QoL) after surgery have not yet been reported. The aim of our systematic review was to assess the effect of perioperative interventions focused on psychological distress on pain, function and QoL after primary TKA for OA of the knee.

## Methods

### Search strategy and study selection

We registered our review protocol at PROSPERO international prospective register of systematic reviews (https://www.crd.york.ac.uk/PROSPERO/) with reference number CRD42016052466 (https://www.crd.york.ac.uk/prospero/display_record.php?ID=CRD42016052466). We performed this systematic review according to the Preferred Reporting Items for Systematic Reviews and Meta-analysis (PRISMA) statement criteria [28].

We performed the literature search according to the guidance of Gasparyan et al. [29]. A professional medical librarian (CdH) identified therapeutic studies (published articles and abstracts of major conferences) exploring the influence of any type of perioperative (before TKA, during surgery, or during postoperative rehabilitation) interventions targeting psychological distress on postoperative outcome (pain, function, and/or QoL) after TKA by searching PubMed, Embase.com, PsycINFO/OVID, CENTRAL, the Cochrane Database of Systematic Reviews, Scopus and Web of Science from inception up to May 26, 2020.

The following terms, including synonyms and closely related words, were used as index terms or free-text words: ‘total knee arthroplasty’ and ‘psychological intervention’. Full search strategies for all the databases are available in Supplementary Appendix 1. Duplicate articles were excluded.

Selection of articles was limited to adults > 18 years who had undergone a primary total knee replacement for osteoarthritis of the knee. We included different study designs (RCTs, cohorts, quasi-experimental studies) investigating the effect of any intervention targeting psychological distress on postoperative pain, function and/or QoL. Minimum duration of follow-up was not an inclusion criterion with the aim to create a complete overview of all studies that have investigated the effect of perioperative interventions focused on psychological distress on pain, function and/or QoL. Perioperative interventions influencing psychological factors of patients had to be clearly defined. Full-text availability was required. There were no restrictions with respect to language, age, or publication source of the paper.

Exclusion criteria were studies not meeting domain, determinant, or outcome, case reports, descriptive studies (in which there was no control group), non-primary literature studies (letter to the editor, reviews, thesis, expert opinions) and articles with no separated results of patients after TKA and total hip arthroplasty (THA) or other types of surgery if various surgical procedures were analysed.

### Main outcome variables

Two authors (JS & GO) independently screened articles for title and abstract and thereafter full text if the abstract potentially met the inclusion criteria. Subsequently, the authors (JS & GO) individually extracted information regarding study design, baseline patient characteristics, baseline clinical findings, follow-up, number of patients initially included in the study, the number of patients available for follow-up and data regarding the primary outcomes of the systematic review. When there was disagreement with respect to data extraction, a third author (AH or RP) could make the final decision.

### Quality assessment

We assessed the risk of bias of the included studies using Cochrane Collaboration’s tool for assessing the risk of bias [30]. Using this tool, two authors (JS & GO) independently scored six types of bias (selection bias, performance bias, detection bias, attrition bias, reporting bias, and other types of bias) as low, high, or unclear on potential risk of bias [30].

We used the Grading of Recommendation, Assessment, Development, and Evaluation (GRADE) approach to qualify the overall level of evidence of outcome measures pain, function and/or QoL (https://www.gradeworkinggroup.org/). Using the GRADEpro software (McMaster University, 2015, available from www.gradepro.org), we graded the quality of evidence as high, moderate, low, or very low [31].

### Data analysis

We arranged the studies according to the type of perioperative intervention (music, education, psychotherapy, and remaining) and collected data of the effect of perioperative interventions targeting psychological distress on postoperative clinical outcome measures pain, function, and QoL. Initially, our intention was to pool data to perform a meta-analysis.

## Results

**Fig. 1:**
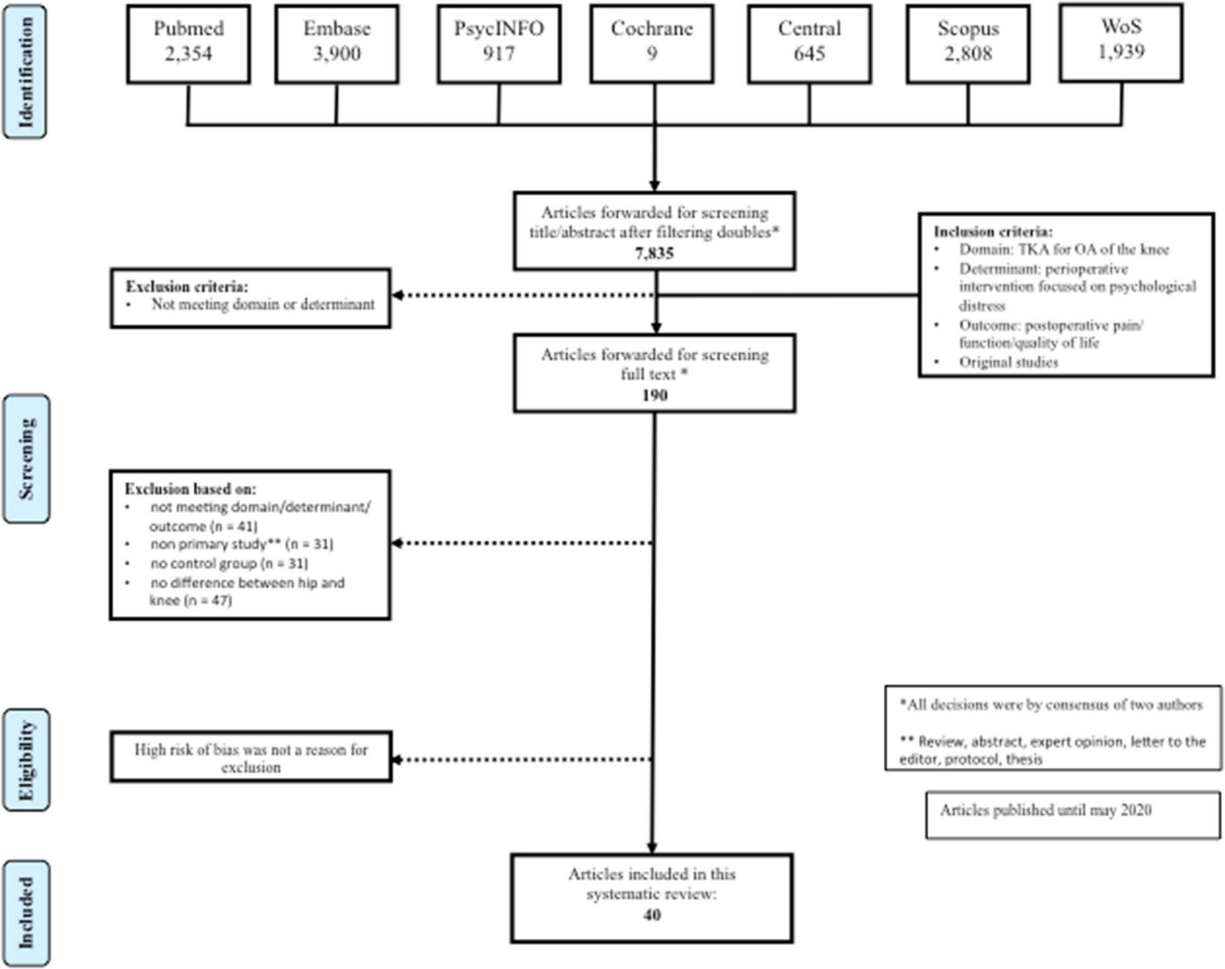
Search strategy and article selection

The search strategy and article selection of articles published from 1964 to 26 May 2020 are shown in the flowchart (Fig. 1). Out of 7835 articles remaining after deduplication, we included 40 studies of which 22 RCTs (one randomised controlled pilot study), 10 cohort studies, and 8 quasi-experimental studies with a total number of 3846 patients.

### Interventions

A description of the interventions in the experimental and the control groups and the time at which the interventions were applied are presented in Table 1.

**Table 1:** Overview of included studies

Type of intervention	Study	Description of intervention	When was the intervention applied?
Music	Allred [32] Prospective cohort	I: Easy-listening music with headphones for 20 min	Before and after their first ambulation at the first postoperative day
		C: 20-min quiet rest period	
	Aris [33] RCT	I: Additional relaxing music therapy during recovery (< 60 beats per minute)	During recovery
		C: Usual care	
	Chen [34] RCT	I: Five compositions of 30 min soothing piano and Chinese violin music (60–80 beats per minute)	Ward before surgery, in the waiting area of the surgical room and twice during postoperative recovery
		C: No music	
	Hsu [35] Prospective cohort	I: Slow relaxing music with slow tempo, low tone and soft melody	Once a day at the 10 a.m. continuous passive motion (CPM) session on the first and second postoperative day
		C: No music, required to rest in bed	
	Hsu [36] Single-group QES	I: Music for 10 min before receiving CPM until the end of the CPM session	During CMP the first and second days after surgery
		C: Rest in bed for 10 min before CPM began	
	Keshmiri [37] RCT	I1: Isolation of noice by soundproof headphones in conjunction to disposable earplugs	During surgery, after the effect of sedative (Propofol) was applied
		I2: Music of patients' choice with headphones	
		C: No isolation of noise or music	
	Leonard [38] RCT	I: Co-treatment session that used live music to support exercise	Postsurgery, after admission to the inpatient rehabilitation unit
		C: Physiotherapy without music	
	Santhna [39] QES	I: Music for five days post-operatively and analgesics	5 days postoperatively
		C: No music, only pharmacological intervention	
	Simcock [40] RCT	I: Music of patients' choice with headphones	During surgery, after a spinal-epidural anaesthesia and sedation with propofol
		C: White noise emanating from the headphones	
Education	Atabaki [41] RCT	I: Educational intervention presented as a combination of lecture, group discussion, individual education, questions and answers	Four perioperative stages (one day before surgery, 24 h and 48 h later, upon discharge from the hospital)
		C: Usual care	
	Aytekin [42] Prospective cohort	I: Education (about OA, joint protection, home safety, and TKA) and home-based exercise	During 12 weeks before the operation
		C: No additional training program, usual care	
	Chen [43] QES	I: Cognitive-behavioural educational intervention (pamphlet, CD and oral instructions)	Before surgery after hospitalisation and 1 days postsurgery
		C Routine care and usual instructions delivered orally	
	Huang [44] RCT	I: 40-min preoperative home rehabilitation education program by a physiotherapist	2–4 weeks prior to admission
		C: No education program	
	Huang [45] RCT	I: Traditional education, telephone education and mobile education	Following surgery
		C: Traditional face-to-face and telephone education	
	Lee [46] RCPS	I1: Psychoeducation on CPSP and prerecorded hypnotic intervention using audiotapes	One delivered before and another delivered at least 24 h after surgery
		I2: Psychoeducation on CPSP and diaphragmatic breathing relaxation exercise	
		C: Usual care	
	Lin [47] QES	I: One-to-one less than 30 min preadmission preoperative teaching*	Preadmission preoperative
		C: Postadmission preoperative teaching and no video	
	Louw [48] CCTWAA	I: Education program and an additional 30-min group pain neuroscience education session	Before surgery
		C: Only education program	
	Malletschek [49] RCT	I: Additional pain psychoeducation over at least 45 min	3–6 days after TKA
		C: Usual care	
	Moulton [50] Prospective cohort	I: Joint school by members of a multidisciplinary group explaining the process of the surgery	Preoperative for 2 h
		C: No joint school	
	Piva [51] RCT	I: Interactive education to promote physical activity and healthy eating	During 3 months postoperative: 2 lectures during the first postoperative week and mini-sessions of physical activity promotion in the subsequent weeks
		C: No education	
	Reslan [52] QES	I: One to one intervention (30–40 min) including education and exercise training by a nurse	Prior to surgery
		C: Standard hospital care	
	Timmers [53] RCT	I: Day-to-day postoperative care information related to topics such as pain, physiotherapy exercises, wound care, and daily self-care activities through an application	During the 28-day period after discharge
		C: Only weekly, basic information	
	Wilson [54] RCT	I: Usual teaching and preoperative educational intervention**	Teaching session and booklet within 4 weeks prior to surgery Phone call during a week before surgery
		C: Usual teaching	
	Yajnik [55] Retrospective cohort	I: Pain management educational card***	Prior to peripheral nerve block placement on the day of surgery, at the time of ward admission by the bedside nurse and once daily during rounds
		C: Before implementation of pain management educational card	
Psychotherapy	Birch [56] RCT	I: CBT based pain education of approximately 45 min delivered by 2 physiotherapists	3 sessions preoperatively and 4 sessions postoperatively (2 weeks before surgery until 3 months after surgery)
		C: Usual care	
	Cai [57] RCT	I: CBT	After TKA
		C: No CBT	
	Cai [58] RCT	I: Individually tailored CBT by a physiotherapist and a psychologist	During 4 weeks after surgery
		C: No CBT	
	Das Nair [59] RCT	I: 10 sessions of CBT during hour-long sessions by one or two psychologists	During waiting time for surgery
		C: No CBT	
	Harnirattisai [60] QES	I: 25-min sessions of nurse-patient interaction and discussion****	At the fourth postoperative day and two weeks after surgery
		C: No behavioural change intervention	
	Jacobson [61] RCT	I: 19- to 21- minute audio recordings of guided imagery$ scripts designed for TKA patients	Every day for two weeks before surgery and three weeks after surgery
		C: Commercially available 17- to 21-min audio recordings	
	Riddle [62] QES	I: Intervention delivered by trained psychologists#	During 8 weekly sessions from approximately one month prior to surgery to one month after surgery
		C: No intervention	
	Riddle [63] RCT	I1: Eight 50-min sessions of 1-on-1 pain coping skills training	Approximately 2 weeks preoperatively to approximately 6 weeks postoperatively
		I2: Eight 50-min sessions of 1-on-1 arthritis education by registered nurses	
		C: Usual care	
	Russo [64] RCT	I: Video according to the Videoinsight Methods^ principles	Three times a week during the first 3 months after surgery
		C: No video	
	Tristaino [23] Prospective cohort	I: Four psychologist-patient sessions of 30 min focusing on defining the psychological themes and concepts on which to focus the activity	One before surgery, two during postoperative hospital stay and one during rehabilitation
		C: Standard of care	
Remaining	Baldwin [66] RCT	I1: Three or four 30-min Reiki treatments provided by three expert Reiki professionals	During the hospital stay
		I2: Standard of care and three or four sham Reiki session delivered by non-trained people	
		C: Standard of care and sessions of “quiet time”	
	Christiansen [67] RCT	I: Standard of care rehabilitation plus weight baring biofeedback training	On the morning before surgery (20 min) and after admission to the post anaesthesia care unit (30 min) and 20 min at the first, second and third postoperative day
		C:Standard of care rehabilitation alone	
	Hiraga [68] NRCT	I: Occupational therapy & self-monitoring using a diary	From 1 to 2 weeks postoperatively
		C: Occupational therapy only	
	Koo [69] RCT	I: Enhanced reality analgesia	Shortly after physiotherapy for 5 times a week, for 2 weeks
		C: No enhanced reality analgesia	Shortly after physiotherapy for 5 times a week, for 1 week
	Notte [70] Prospective cohort	I: Weight bearing (WB) biofeedback-assisted progressive muscle relaxation training sessions using a Nintendo Wii fit Plus game and associated Wii balance board	Twice weekly at home for 6 weeks after surgery
		C: Standard of care	
	Wang [71] QES	I: CPM therapy and 30-min biofeedback relaxation training	One day before surgery and twice a day on the five first postoperative days, concurrent with CPM therapy
		C: Only CPM therapy	

*I* intervention group, *C* control group, *RCT* randomised controlled trial, *CPM* continuous passive motion, *QES* quasi-experimental study, *OA* osteoarthritis, *TKA* total knee arthroplasty, *CD* compact disk, *RCPS* randomized controlled pilot study, *CPSP* chronic postsurgical pain, *CCTWAA* controlled clinical trial with alternating allocation, *CBT* cognitive behavioural therapy, *NRCT* non-randomised controlled trial

^*^Preoperative education about care pathway, knee surgery, pain management, expected discharge goals and in-patient and out-patient arthroplasty rehabilitation by an educational nurse and a booklet

^**^Preadmission preoperative teaching with an instruction booklet during a preoperative outpatient clinic visit. Upon admission to the hospital, they were presented with an educational videotape

^***^A booklet containing symptom management after TKA, an individual teaching session, and a follow-up support call by the principal investigator

^****^25-Min sessions of nurse-patient interaction and discussion regarding specific exercises and physical activity, self-monitoring, goal setting, family support and encouragement, and information prompting

^$^Guided imagery is a widely used mind–body intervention by the generation of self- or practitioner-guided positive sensory and affective mental images to promote health changes in the body, reducing anxiety and stress, and evoking psychological and physiologic relaxation [61]

^#^Intervention addressed to the recovery of physical function, the concerns during the recovery period and strategies for coping with pain after the operation delivered by trained therapists

^The video was established to produce positive and therapeutic insight, according to the Videoinsight Methods principles [65]

### Music

Nine studies examined the effect of perioperative listening to music on postoperative outcome. Eight of these studies [32–34, 36–40] assessed the effect of music on pain and three [35, 36, 40] on function. Music was offered at different time points and different types of music were provided.

### Education

The effect of education on postoperative outcome was investigated in fifteen studies in which the time of education varied from 12 weeks before surgery to 3 months after surgery (Table 1).

### Psychotherapy

Psychological therapies provided with direct support from a professional were examined by eight studies. The patients in the RCTs of Jacobson et al. [61] and Russo et al. [64], who also received psychological therapy, received their psychological intervention by audio recordings, or watching a video instead of direct contact with a health care professional.

### Other/remaining interventions

Four remaining interventions (Reiki, biofeedback relaxing training and enhanced reality analgesia, self-monitoring using a diary), applied to six studies, could not be allocated to the music, educational, or psychological therapy intervention groups and were, therefore, classified as remaining interventions (Table 1).

### Outcomes

Outcome measures pain, function, and/or QoL were assessed in 22 RCTs (one randomised controlled pilot study), 10 cohort studies, and 8 quasi-experimental studies. Mean age of the patients ranged from 61.7 to 74.1 years and duration of follow-up ranged between 60 min and 2 years. Due to the heterogeneity of the type of studies, interventions, outcome measures and follow-up there was no possibility to pool data to perform a meta-analysis.

### Pain

34 studies examined the influence of a perioperative intervention targeting psychological distress on clinical outcome pain after the TKA. Many different scoring systems were used to score postoperative pain and eight studies assessed pain medication use as an outcome measure for pain (Table 2).

**Table 2:** The influence of perioperative interventions targeting psychological distress on pain after the TKA

Type of intervention	Study	Nr TKA		Females (%)	Age mean ± SD	Follow-up	Outcome score (pain)	I score ± SD	C score ± SD	Statistically significance at latest follow-up
Music	Allred 2010	T	56	31 (55.4)	63.9 (64-84)*	6 hours	VAS	41.2 ± 25.8	45.1 ± 31.2	*P* = 0.337
		I	28				MPQ	15.9 ± 10.6	14.9 ± 12.3	*P* = na, no statistical analysis between groups
		C	28				Opioid use (morphine or dilaudid)	na	na	*P* = 0.388 and *P* = 0.152 (regarding which oral medication)
	Aris 2019	T	56			60 minutes	VAS	0 (24.39 **	1.5 (32.61)**	*P* = 0.045
		I	28	19 (67.9)	63.71±11.005					
		C	28	19 (67.9)	64.50±8.851					
	Chen 2015	T	30	20 (66.7)	68 (53-85)*	Postoperative days	VAS (recovery)	3.22 ± 0.22***	3.00 ± 0.25***	*P* = 0.50
		I	15				VAS (ward)	3.07 ± 0.26***	2.87 ± 0.18***	*P* = 0.53
		C	15				Opioid use (parenteral morphine, meperidine, fentanyl in PO recovery)	7.39 ± 2.66	6.86 ± 2.29	*P* = 0.57
							Opioid use (parenteral morphine, meperidine, fentanyl in the ward)	12.04 ± 14.43	12.90 ± 8.05	*P* = 0.89
	Hsu 2019	T	49	34 (69.4)	73.9 ± 7.5	2 days	NRS	0.06 ± 0.24	2.14 ± 1.10	*P* < 0.01
		I	49							
		C	49							
	Keshmiri 2014	T	83	52 (62.7)	68.7 ± 0.96	2-7 days	VAS (day 1-3)	1.33 ± 0.11 (I1) & 1.44 ± 0.13 (I2)	1.49 ± 0.13	*P* = 0.718
		I1	28				VAS (day 4-7)	0.9 ± 0.15 (I1) & 0.81 ± 0.13 (I2)	1.23 ± 0.19	*P* = 0.330
		I2	27				VAS (day 17)	1.09 ± 0.12 (I1) & 1.08 ± 0.11 (I2)	1.34 ± 0.14	*P* = 0.435
		C	28				Days of pain catheter duration (type of pain medication na)	3.43 ± 0.11 (I1) 7 3.48 ± 0.12 (I2)	3.36 ± 0.19	*P* = 0.452
	Leonard 2019	T	32			Postoperative days	NRS	5.44 ± 3.2	5.56 ± 2.52	"No significant difference"
		I	16	11 (68.8)	67.9 (45-87)*		Observational coding for pain	3.06 ± 3.13	2.31 ± 2.36	*P* = 0.02
		C	16	12 (75)	67.6 (53-80)*					
	Santhna 2015	T	40	14 (70) 18 (90)	63.80±5.64 64.90±6.94	5 (days)	PRI	11.78^	29.23^	*P* = 0.00
		I	20							
		C	20				VAS	14.20^	26.80^	*P* = 0.00
							PPI	15.00^	26.00^	*P* = 0.001
							Paracetamol	16000mg^^	17000 mg^^	*P* > 0.05
							Celecoxib	600 mg^^	1600 mg^^	*P* > 0.05
							Tramadol	125 mg^^	225 mg^^	*P* > 0.05
	Simcock 2008	T	30	18 (60)	67.3±9.1	24 hours	VAS (3 hours PO)	3.87 ± 3.44	1.47 ± 1.39	*P* = 0.01
		I	15				VAS (6 hours PO)	5.26 ± 3.04	3.38 ± 2.48	*P* = 0.075
		C	15				VAS (24 hours PO)	4.03 ± 2.89	2.41 ± 1.67	*P* = 0.04
Education	Atabaki 2019	T	56			6 (weeks)	WOMAC	40.47 ± 10.47	57.29 ± 7.51	*P* = 0.001
		I	48	46 (95.8)	65.39 ± 5.08					
		C	48	41 (85.4)	63.83 ± 5.14					
	Aytekin 2019	T	44			6 months	VASpr	0.4 ± 0.9	0.8 ± 1.1	"no significant difference between groups"
		I	23	18 (78.3)	67.8 ± 6.3		VASpa	1.5 ± 1.5	2.3 ± 2.3	"no significant difference between groups"
		C	21	18(85.7)	69.7 ± 6.4		KOOSpain	87.9 ± 15.4	92.7 ± 8.3	"no significant difference between groups"
	Chen 2014	T	92	63 (68.5)	69.26 ± 9.025	5 days	NRS (worst pain)	4.89 ± 2.82	5.57 ± 2.84	*P* = 0.308
		I	42				NRS (average pain)	2.38 ± 1.97	2.43 ± 2.03	*P* = 0.916
		C	50				NRS (current pain)	2.46 ± 2.31	2.57 ± 2.26	*P* = 0.836
	Huang 2011	T	242	174 (71.6)	70.2 ± 7.3	5 days	VAS	2.4 ± 0.7	2.5 ± 0.6	*P* = 0.686
		I	125							
		C	117							
	Louw 2019	T	103			6 (months)	NRS	na	na	*P* = 0.386
		I	49	32 (65.3%)	74.1 ± 9.5		Morfine	2601.62 ± 1103.90	2734.02 ± 1324.60	*P* = 0.635
		C	54	23 (51.9)	69.6 ± 10.6					
	Mallet-schek 2019	T	75	47 (62.7)	59-78*	3 months	KOOSpain	na	na	*P* = 0.01
		I	37							
		C	38							
	Lee 2019	T	24			6 months	NRS	1.40 ± 0.89 (I1) & 1.73 ± 1.40 (I2)	2.23 ± 1.41	HYP vs. control: *P* = 0.134 and P = 0.038 (when controlled for covariates) MET vs. Control: *PP* = 0.975
		I1	8	7 (87.5)	65.63 ± 9.27					
		I2	8	7 (87.5)	56.25 ± 11.22					
		C	8	8 (100)	67.88 ± 10.38					
	Moulton 2017	T	563	na	70.1 ± na	2 years	OKS (6 months PO)	28.71 ± na	31.60 ± na	*P* = 0.251
		I	503				OKS (2 years PO)	30.17 ± na	33.26 ± na	*P*= 0.440
		C	60							
	Piva 2017	T	44	31 (70.5)		6 months	WOMAC pain	min 1.7 (95% CI -3.0,-4.0) ^^^	min 0.3 (95% CI - 1.5, 1.0) ^^^	*P* = 0.035
		I	22		68.1 ± 7.5					
		C	22		68.3 ± 5.5					
	Reslan 2018	T	60		na	4 weeks	HSSpain	22.83 ± 4.78	19.18 ± 5.14	*PP* = 0.001
		I	30	19 (63.6)						
		C	30	17 (56.7)						
	Timmers 2019	T	213			4 weeks after discharge	NRS at rest	3.45^	4.59^	*PP* = 0.001
		I	114	74 (64.9)	64.74 ± 7.57		NRS activity	3.99^	5.08^	*P* < 0.001
		C	99	60 (60.6)	65.63 ± 7.90		NRS at night	4.18^	5.21^	*P* = 0.003
	Wilson 2016	T	143	89 (62.6)		3 days	BPI-I	24.4 ± 14.4	22.4 ± 15.1	*PP* = 0.45
		I	73		67 ± 8		NRS (rest)	2.8 ± 2.5	2.8 ± 2.7	*P* = 0.70
		C	70		66 ± 8		NRS (moving)	5.4 ± 3.0	6.1 ± 2.5	*P* = 0.20
							NRS worst pain last 24 hours)	7.0 ± 2.4	7.0 ± 2.3	P = 0.87
							Opioid use (morphine, hydroporphone, oxycodon, codeine)	40 (45)*^	40 (42)*^	"no difference between groups in daily 24-hours opioid administration"
	Yajnik 2018	T	40	3 (7.5)	68 (46-80)*	2 days	Opioid use (morphine, MME PO day 1 and 2)	38 (1-117)*	72 (32-285)*	*P* = 0.001
		I	20				Minimum pain (patients’ verbal rating 0–10) 1 day PO	0 (0 - 3)*	0 (0 - 6)*	*P* = 0.151
		C	20				Maximum pain (patients’ verbal rating 0–10) 1 day PO	4 (2 - 9)*	8 (1 - 10)*	*P* = 0.114
Psycho-therapy	Birch 2019	T	60			1 (year)	VAS activity	12 (5-18)^^^	9 (3-15) ^^^	*P* = NS
		I	31	22 (33)	66 ± 9		VAS rest	7 (1–12)^^^	6 (1–12) ^^^	*P* = NS
		C	29	18 (27)	66 ± 10					
	Cai 2017	T	108			6 months	KSS	82.61 ± 6.38	73.30 ± 8.45	*P* < 0.01
		I	54	31 (57.4)	62.42 ± 6.59					
		C	54	34 (63.0)	63.94 ± 6.58					
	Cai 2018	T	100	62 (55.9)		6 months	NRS	5.63 ± 0.73	6.27 ± 0.86	time effects: *P* < .001); group effects: *P* = 0.003); group-by-time interaction: *P* = 0.080
		I	50		65.26 ± 8.30					
		C	50		66.18 ± 7.04					
	Das Nair 2018	T	50	23 (46)			WOMAC pain	6.5 ± 3.6	7.5 ± 2.3	*P* = 0.40
		I	25		65.7 ± 8.6		ICOAP constant pain (item 1-5)	6.4 ± 4.4	6.2 ± 3.2	*P* = 0.99
		C	25		66.7 ± 9.9		ICOAP constant pain (item 1, 3, 4, 5)	4.8 ± 3.7	5.1 ± 3.0	*P* = 0.82
							ICOAP constant pain (converted rasch score item 1, 3, 4, 5)	5.5 ± 4.1	6.0 ± 3.2	*P* = 0.75
							ICOAP intermittent pain (item 6-11)	8.5 ± 5.6	10.2 ± 4.5	*P* = 0.43
							ICOAP intermittent pain (item 6, 7, 10, 11)	5.7 ± 3.8	7.1 ± 3.3	*P* = 0.33
							ICOAP intermittent pain (converted rasch score item 6, 7, 10 11)	5.5 ± 3.4	6.7 ± 3.0	*P* = 0.34
	Jacobson 2016	T	58	51 (62.2)	65 (41-81)*	6 months	WOMAC pain	2.7 ± 3.1	3.5 ± 3.3	*P* < 0.001
		I	29				VAS daily pain	na	na	*P* not available at 6 months postoperatively
		C	29							
	Riddle 2011	T	63	45 (71.4)		2 months	WOMAC pain	6.0 ± 4.1	8.6 ± 3.7	*P* = 0.017
		I	18		63.8 ± 11.5					
		C	45		60.8 ± 9.9					
	Riddle 2019	T	402			12 months	WOMAC pain	3.3 (95% CI 2.5, 4.2) (I1) & 3.0 (95% CI 2.1, 3.8) (I2)^^^	2.9 (95% CI 2.0, 3.8)^^^	*P*= 0.60
		I1	130	94 (72.3)	62.6 ± 7.9		NRS	1.8 (95% CI 1.2, 2.4) (I1) & 2.0 (95% CI 1.3,2.6) (I2) ^^^	1.7 (95% CI 1.1, 2.2) ^^^	*P* = na
		I2	135	85 (63.0)	64.2 ± 8.5					
		C	137	88 (64.2)	62.7 ± 7.7					
	Tristaino 2015	T	64	44 (62.0)		4 months	SF-36 bodily pain	70.1 ± 21.5	67.8 ± 26.8	*P* = 0.715
		I	33		64.2 ± 8.6					
		C	31		66.1 ± 6.6					
Remaining	Baldwin 2017	T	56	na	na	72 hours	VAS	na	na	"Reiki significant pain reduction (*P* = 0.003), Sham Reiki and SOC no significant reduction"
		I1	25				Opioid use (oxycontin, oxycodone, morphine)	na	na	*P* = na, not mentioned in significant results)
		I2	12							
		C	19							
	Hiraga 2019	T	41			4 weeks	NRS rest	1.3 ± 0.4	1.2 ± 0.4	*P* = 0.965
		I	20	16 (80)	76.4 ± 7.1		NRS walk	1.3 ± 0.2	3.2 ± 0.6	*P*= 0.017
		C	21	19 (90.4)	76.6 ± 5.5					
	Koo 2018	T	120			5 weeks	VAS	na (figure)	na (figure)	"No signicance was found in VAS analyses between the groups"
		I	60	17 (28.3)	65.00 ± 6.97					
		C	60	15 (25)	63.71 ± 5.09					
	Notte 2016	T	43	na	na	3 days postoperatively	NRS	na	na	*P* = 0.000 (1, 2, 3 days PO)
		I	23				Opioid use (type of opioid na)	na	na	*P* = 0.92
		C	20							
	Wang 2015	T	66	23 (34.9)	73.5 ± 9.5	5 days	NRS	3.36 ± 1.47	4.23 ± 1.67	*P* < 0.001
		I	33				Opioid use (pethidine PO day 5)	1 (3.2)	0 (0.0)	*P* = 0.49
		C	33				PMU (Acetaminophen or COX-2 inhibitor + pethidine or tramadol PO day 5)	24 (77.4)	21 (63.6)	*P* = 0.27

*Nr* number;* TKA* total knee arthroplasty;* SD* standard deviation;* I* intervention group;* C* control group;* T* total study group;* VAS* visual analog scale;* P P* value;* MPQ* short form McGill pain questionnaire; na: not available;* PO* postoperative;* NRS* numeric rating score;* PRI* Pain Rating intensity;* PPI* Present Pain Intensity;* mg* milligram;* WOMAC* Western Ontario and McMaster universities osteoarthritis index;* VASpr* visual analog scale pain resting;* VASpa* visual analog scale pain acitivity;* KOOSpain* pain subscale of the knee injury and osteoarthritis outcome score;* HYP* hypnotic intervention;* MET* minimal-effect treatment;* OKS* Oxford knee score;* 95% CI* 95% confidence interval;* HSS* hospital for special surgery;* BPI-I* Brief Pain Inventory interference;* MME* Morphine Milligram Equivalents;* NS* not significant;* KSS* knee society score;* ICOAP* Intermittent and Constant Osteoarthritis Pain scale;* SF-36* Short Form-36;* SOC* stand of care;* PMU* pain medication use;* COX-2* cyclooxygenase-2

Instead of mean and SD: *median (range), **median and mean rank, ***mean and standard error, ^mean rank only, ^^median only, ^^^mean estimate with the 95% CI in parentheses, *^median (interquartile range) instead of mean and SD

As shown in Table 2, patients in the intervention groups had significant better postoperative pain scores or declined prescriptions of opioids in 20 studies. Therapies applied in these studies were music during surgery [40] or after surgery [33, 36, 38, 39], education [41, 49, 51–53, 55], cognitive behavioural therapy [57, 58], guided imagery [61], pain coping skills training [62], Reiki therapy [66, 70], occupational therapy in combination with self-monitoring using a diary [68], weight-bearing biofeedback training [67] and biofeedback-assisted progressive muscle relaxing training [71]. The remaining 14 studies did not show a significant effect on any of the pain-related outcome measures or pain medication use at the latest follow-up when using a perioperative intervention focused on psychological distress in conjunction to TKA.

### Function

A total of 29 studies examined the effect of an intervention targeting psychological distress on function after the TKA (Table 3).

**Table 3:** The influence of perioperative interventions targeting psychological distress on function after the TKA

Type of intervention	Study	Nr TKA		Females (%)	Age mean ± SD		Follow-up	Outcome score (function)	I score ± SD	C score ± SD	Statistically significance at latest follow-up
Music	Hsu [35]	T	91	67 (73.6)			2 days	CPM angles 1 day PO	24.29 ± 5.00	12.98 ± 4.43	*P* < 0.01
		I	49		73.9 ± 7.5			CPM angles 2 days PO	21.22 ± 2.98	16.07 ± 4.49	*P* < 0.01
		C	42		71.33 ± 8.45			Active knee flexion ROM 2 days PO	106.22 ± 6.17	95.00 ± 6.80	*P* < 0.01
	Hsu [36]	T	49				2 days	Increased degree of knee flexion during CPM	21.22 ± 2.98	10.02 ± 3.03	*P* < 0.01
		I	49	34 (69.4)	73.9 ± 7.5						
		C	49	34 (69.4)	73.9 ± 7.5						
	Leonard [38]	T	32				Postoperative days	Observational coding for pedalling adherence	7.81 ± 0.40	7.44 ± 1.21	"No significant difference"
		I	16	11 (68.8)	67.9 (45–87)*						
		C	16	12 (75)	67.6 (53–80)*						
Education	Atabaki [41]	T	96				6 weeks	WOMAC stiffness	19.53 ± 12.34	41.66 ± 10.09	*P* = 0.001
		I	48	46 (95.8)	65.39 ± 5.08			WOMAC performance difficulty	43.48 ± 7.96	55.82 ± 4.30	*P* = 0.001
		C	48	41 (85.4)	63.83 ± 5.14						
	Aytekin [42]	T	44				6 months	KOOS total	82.2 ± 16.1	85.5 ± 9.5	"No significant difference between groups"
		I	23	18 (78.3)	67.8 ± 6.3			KOOSdaily living activities	87.2 ± 18.3	91.1 ± 9.2	"No significant difference between groups"
		C	21	18(85.7)	69.7 ± 6.4			KOOSsports	52.8 ± 24.4	56.1 ± 13.1	"No significant difference between groups"
	Chen [43]	T	92	63 (68.5)	69.26 ± 9.025		5 days	Overall rating of nine physical function items	12.38 ± 2.806	12.05 ± 3.682	*P* = 0.625
		I	42					Ankle pumping	1.55 ± 0.39	1.54 ± 0.44	*P* = 0.927
		C	50					Quadriceps setting	0.17 ± 0.39	0.23 ± 0.43	*P* = 0.518
								Knee flexion/extension	0.44 ± 0.53	0.69 ± 0.66	*P* = 0.062
								Straight-leg raises	1.22 ± 2.58	0.64 ± 0.56	*P* = 0.000
								MPOAL	3.71 ± 0.622	3.08 ± 1.090	*P* = 0.004
	Huang 2011	T	242	174 (71.6)	70.2 ± 7.3		5 days	Ability to walk during discharge	85.7 ± na	81.2 ± na	*P* = 0.343
		I	125					ROM	76 ± 22	74 ± 20	*P* = 0.582
		C	117								
	Huang [45]	T	150	102 (68.0)			3 months	ROM ITT ROM PP	110.6 ± 6.68 110.0 ± 6.33	105.00 ± 8.82 103.26 ± 7.57	*P* < 0.001 *P* < 0.001
		I	75		62.42 ± 6.59						
		C	75		63.94 ± 6.58						
	Lin [47]	T	60	31 (51.7)	68.6 ± na			EPC	14.93 ± na	8.87 ± na	*P* < 0.05
		I	30					Knee flexion	77.84 ± na	70.16 ± na	*P* = 0.013
		C	30					Ambulation ability	na	na	"The differences between groups were not significant"
	Louw [48]	T	101				6 months	WOMAC	na	na	*P* = 0.222
		I	49	32 (65.3)	74.1 ± 9.5						
		C	54	23 (51.9)	69.6 ± 10.6						
	Malletschek 2019	T	75	47 (62.7)	59 – 78**		3 months	KSS	na	na	*P* = 0.08
		I	37								
		C	38								
	Moulton [50]	T	563	na	70.1 ± na		2 years	OKS (6 months PO)	28.71 ± na	31.60 ± na	*P* = 0.251 (6 months)
		I	503								
		C	60								
								OKS (2 years PO)	30.17 ± na	33.26 ± na	*P* = 0.440 (2 years)
	Piva [51]	T	44	31 (70.5)			6 months	SF-36 PF	76.7 ± 16.1	70.3 ± 24.2	*P* = 0.017
		I	22		68.1 ± 7.5			Single-leg stance test	16.1 ± 9.6	17.4 ± 9.8	*P* = 0.037
		C	22		68.3 ± 5.5			WOMAC PF	11.8 ± 6.7	12.8 ± 10.8	*P* = 0.558
								Stair-climb	14.3 ± 4.1	15.6 ± 7.4	*P* = 0.054
								Chair-stand	12.2 ± 2.8	13.7 ± 7.5	*P* = 0.149
								6-Min walk	472.6 ± 86.5	518.0 ± 103.3	*P* = 0.638
								Gait speed	1.14 ± 0.16	1.18 ± 0.24	*P* = 0.790
								Daily activity	152.5 ± 93.3	174.9 ± 126.1	*P* = 0.279
	Reslan [52]	T	60				4 weeks	HSSfunction	15.73 ± 3.49	13.92 ± 3.35	*P* = 0.026
		I	30	19 (63.6)	na			HSSrom	17.04 ± 2.55	16.53 ± 4.20	*P* = NS
		C	30	17 (56.7)	na			HSSquadriceps muscle strength	9.13 ± 3.81	8.47 ± 2.93	*P* = NS
								HSSflexion deformity	10.02 ± 1.21	8.47 ± 1.93	*P* = 0.007
								HSSinstability	9.89 ± 3.41	8.27 ± 2.89	*P* = 0.049
								LEFS	60.35 ± 11.22	53.83 ± 12.98	*P* = 0.048
	Timmers [53]	T	213				4 weeks after discharge	KOOS	37.61 ± 10.17	43.08 ± 12.96	*P* < 0.001
		I	114	74 (64.9)	64.74 (7.57)			Ability to perform physiotherapy	7.50***	6.88***	*P* = 0.03
		C	99	60 (60.6)	65.63 (7.90)			Ability to perform self-care activities	8.32***	7.64***	*P* = 0.004
	Yajnik 2018	T	40	3 (7.5)	68 (46–80)*		2 days	Maximum ambulation 1 day PO	20 (0–59)^	12 (0–30) ^	*P* = 0.069 (POD 1)
		I	20					Maximum ambulation 2 days PO	46 (6–67)^	38 (0–61) ^	*P* = 0.141 (POD 2)
		C	20								
Psycho-herapy	Birch 2019	T	60				1 year	OKS	33 (29, 27)^^	37 (33, 41)^^	*P* = NS
		I	31	22 (33)	66 (9)			6-Min walk	441 (402,480)^^	406 (367, 446)^^	*P* = NS
		C	29	18 (27)	66 (10)			Sit to stand	12 (11, 14) ^^	11 (95% CI 10,13) ^^	*P* = NS
	Cai [57]	T	108				6 months	KSS	82.61 ± 6.38	73.30 ± 8.45	*P* < 0.01
		I	54	31 (57.4)	62.42 ± 6.59			First time out of bed (hours)	22.13 ± 4.18	36.41 ± 7.31	*P* = < 0.001
		C	54	34 (63.0)	63.94 ± 6.58						
	Cai [58]	T	100	62 (55.9)			6 months	HSS function	80.68 ± 8.02	68.98 ± 8.64	*P* < 0.001 (time interaction), *P* < 0.001 (group interaction), *P* = 0.003 (group-by-time interaction)
		I	50		65.26 ± 8.30						
		C	50		66.18 ± 7.04						
	Das Nair [59]	T	50	23 (46.0)			6 months	WOMAC function	20.9 ± 12.7	32.0 ± 4.8	*P* = 0.009
		I	25		65.7 ± 8.6			WOMAC stiffness	3.2 ± 1.9	4.2 ± 0.9	*P* = 0.11
		C	25		66.7 ± 9.9						
	Harnirattisai [60]	T	63	59 (93.7)	67.88 (60–85)*		6 weeks	PTT total	8.86 ± 1.89	6.43 ± 1.66	*P* = na
		I	42					PPT standing balance	Δ 2.00 ± 1.22^^^	Δ 1.09 ± 1.22^^^	*P* = 0.016
		C	21					PPT walking speed	Δ 1.55 ± 1.02^^^	Δ 0.76 ± 0.83^^^	*P* = 0.004
								PPT chair-stand	Δ 2.36 ± 1.05^^^	Δ 1.33 ± 1.02^^^	*P* < 0.001
								ADL and daily requirements exercise activity	na	na	"There were no significant differences in ADL participation"
	Jacobson [61]	T	58	51 (62.2)	65 (41–81)*		6 months	SF-36 physical	50.4 ± 6.0	47.3 ± 7.5	*P* = na
		I	29					WOMAC stiffness	1.9 ± 1.4	2.1 ± 1.9	*P* = na
		C	29					WOMAC function	7.2 ± 7.1	10.2 ± 10.5	*P* = na
								Gait velocity	na	na	*P* = 0.0154 (group-by-imaging ability interaction)
								Timed walk in seconds	7.4 ± 2.2	8,5 ± 2.3	*P* = na
	Riddle [62]	T	63	45 (71.4)			2 months	WOMAC disability	18.3 ± 12.2	24.1 ± 10.9	*P* = 0.023 (for differences among discharge scores for the 2 groups after adjusting for baseline differences)
		I	18		63.8 ± 11.5						
		C	45		60.8 ± 9.9						
	Riddle [63]	T	402				12 months	WOMACfunction	11.7 (8.6, 14.9) (I1) & 12.2 (9.0, 15.4) (I2)^^	10.5 (7.4, 13.6)^^	*P* > 0.05
		I1	130	94 (72.3)	62.6 ± 7.9			SPPB	8.0 (7.2, 8.7) (I1) & 8.4 (7.6, 9.1) (I2)^^	8.6 (95% CI 7.8, 9.4)^^	*P* > 0.05
		I2	135	85 (63.0)	64.2 ± 8.5						
		C	137	88 (64.2)	62.7 ± 7.7						
	Russo 2016	T	110	na	69.1 ± na		3 months	SF-36 physical	45.6 ± 8.3	46.2 ± 9.9	*P* > 0.01
		I	55					KSS	87.8 ± 9.6	78.3 ± 8.2	*P* = < 0.005
		C	55					WOMAC	79.9 ± 13.0	69.7 ± 9.5	*P* = < 0.005
								VAS functional score	2.8 ± 1.6	4.0 ± 1.5	*P* = < 0.005
	Tristaino 2015	T	44	44 (62.0)			4 months	SF-36 PCS	49.5 ± 6.6	50.9 ± 9.8	*P* = 0.5114
		I	33		64.2 ± 8.6			Days until physiotherapy objective reached	8.1 ± 2.4	8.8 ± 2.3	*P* = 0.2424
		C	31		66.1 ± 6.6						
Remaining	Chistriansen 2015	T	26	13 (50)			26 weeks	FTSST	9.5 ± 2.4	9.6 ± 1.6	*P* = 0.21
		I	13			68.2 ± 8.6		Hip moment (Nm/kg) during FTSST	0.65 ± 0.24	0.63 ± 0.20	*P* = 0.686
		C	13			66.6 ± 8.1		Knee moment (Nm/kg) during FTSST	1.03 ± 0.22	0.97 ± 0.11	*P* = 0.434
								Ankle moment (Nm/kg) during FTSST	0.17 ± 0.16	0.24 ± 0.14	*P* = 0.227
								Walking speed (m/s)	1.29 ± 0.25	1.24 ± 0.13	*P* = 0.68
								Hip moment during walking	0.28 ± 0.19	0.36 ± 0.22	*P* = 0.160
								Knee extension moment during walking	0.61 ± 0.25	0.42 ± 0.44	*P* = 0.008
								Ankle moment during walking	0.09 ± 0.29	0.01 ± 0.19	*P* = 0.877
	Hiraga [68]	T	41				4 weeks	Daily step count	3580.5 ± 1545.2	2088.4 ± 2008.3	*P* = 0.041
		I	20	16 (80)		76.4 ± 7.1		Psychical activity time	1741.4 ± 551.3	731.8 ± 321.1	*P* = 0.000
		C	21	19 (90.4)		76.6 ± 5.5					
	Koo [69]	T	120				5 weeks	WOMAC	14.59 ± 9.14	10.86 ± 10.84	*P* = 0.398
		I	60	17 (28.3)		65.00 ± 6.97		Graded ambulation distance	na	na	*P* = na
		C	60	15 (25)		63.71 ± 5.09		6-Min walk test	407.00 ± 83.62	353.35 ± 82.35	*P* = 0.163
								Timed-stand test	19.29 ± 2.80	19.00 ± 6.16	*P* = 0.967

*Nr* Number, *TKA* total knee arthroplasty, *SD* standard deviation, *I* intervention group, *C* control group, *T* total study group, *CPM* continuous passive motion, *PO* postoperative, *P* *P* value, *ROM* range of motion, *WOMAC* Western Ontario and McMaster Universities osteoarthritis index, *KOOS* Knee Injury and Osteoarthritis Outcome Score, *MPOAL* muscle power of the affected leg, *ITT* intention to treat, *PP* per protocol, *na* not available, *EPC* exercises performance checklist, *KSS* Knee Society Score, *OKS* Oxford knee score, *SF-36 PF* Short Form-36 physical functioning, *HSS* hospital for special surgery knee score, *NS* not significant, *LEFS* lower extremity functional scale, *POD* postoperative day, *PPT* physical performance test, *ADL* activities of daily living, *SPPB* short physical performance battery, *VAS* visual analog scale, *PCS* physical component scale, *FTSST* five-time sit-to-stand test, *Nm/kg* Newtonmeter/kilogram, *m/s* metre per second

Instead of mean and SD

^*^Mean (range)

**Range only

***Mean only

^Median (10th–90th percentiles)

^^Mean estimate with the 95% CI parentheses

^^^Mean change score baseline—6 weeks postoperative

As shown in Table 3, function was significantly improved by perioperative interventions in 18 studies. Pain coping skills training [62], audiorecording guided imagery scripts [61], video promoting self-confidence and psychological support [64], music [35, 36], occupational therapy in combination with self-monitoring using a diary [68], various types of education [41, 43, 45, 47, 51–53], weight-bearing biofeedback training [67], and psychological therapies (behavioural change intervention [60] and cognitive behavioural therapy [57–59]) positively affected any, but not all, of the functional outcome measures after TKA. In the most recent study by Riddle et al. [63], patients receiving pain coping skills training did not have significantly better scores on WOMAC function and the short physical performance battery. Other types of education [42, 44, 48–50, 55], music during physiotherapy [38], enhanced reality analgesia [69], cognitive behavioural therapy delivered by physiotherapists [56], and psychological support from a professional psychologist [23] did also not affect any of the functional outcome measures after TKA.

### QoL

Two recent studies [49, 53] examined the effect a perioperative intervention on QoL (Table 4). Patients receiving postoperative day-to-day education through an app seemed to report significantly better QoL compared to patients who received usual care [53]. Additional psychoeducation did not significantly improve QoL [49].

**Table 4:** The influence of perioperative interventions targeting psychological distress on QoL after the TKA

Type of intervention	Study	Nr TKA		Females (%)	Age mean ± SD	Follow-up	Outcome score (QoL)	I score ± SD I score ± SD	C score ± SD	Statistically significance at latest follow-up
Education	Malletscheck 2019	T	75	47 (62.7)	59–78*	3 months	KOOS QoL	na	na	*P* = NS
		I	37							
		C	38							
	Timmers 2019	T	213			4 weeks after discharge	EQ-5D	0.76 ± 0.16	0.67 ± 0.25	P < 0.001
		I	114	74 (64.9)	64.74 ± 7.57					
		C	99	60 (60.6)	65.63 ± 7.90					

*Nr* Number, *TKA* total knee arthroplasty, *SD* standard deviation, *QoL* quality of life, *I* intervention group, *C* control group, *T* total study group, *KOOS* Knee Injury and Osteoarthritis Outcome Score, *na* not available, *P* *P* value, *NS* not significant, *EQ-5D* EuroQOL Five-Dimensional Questionnaire

Instead of mean and SD

^*^Range

### Quality assessment

**Fig. 2:**
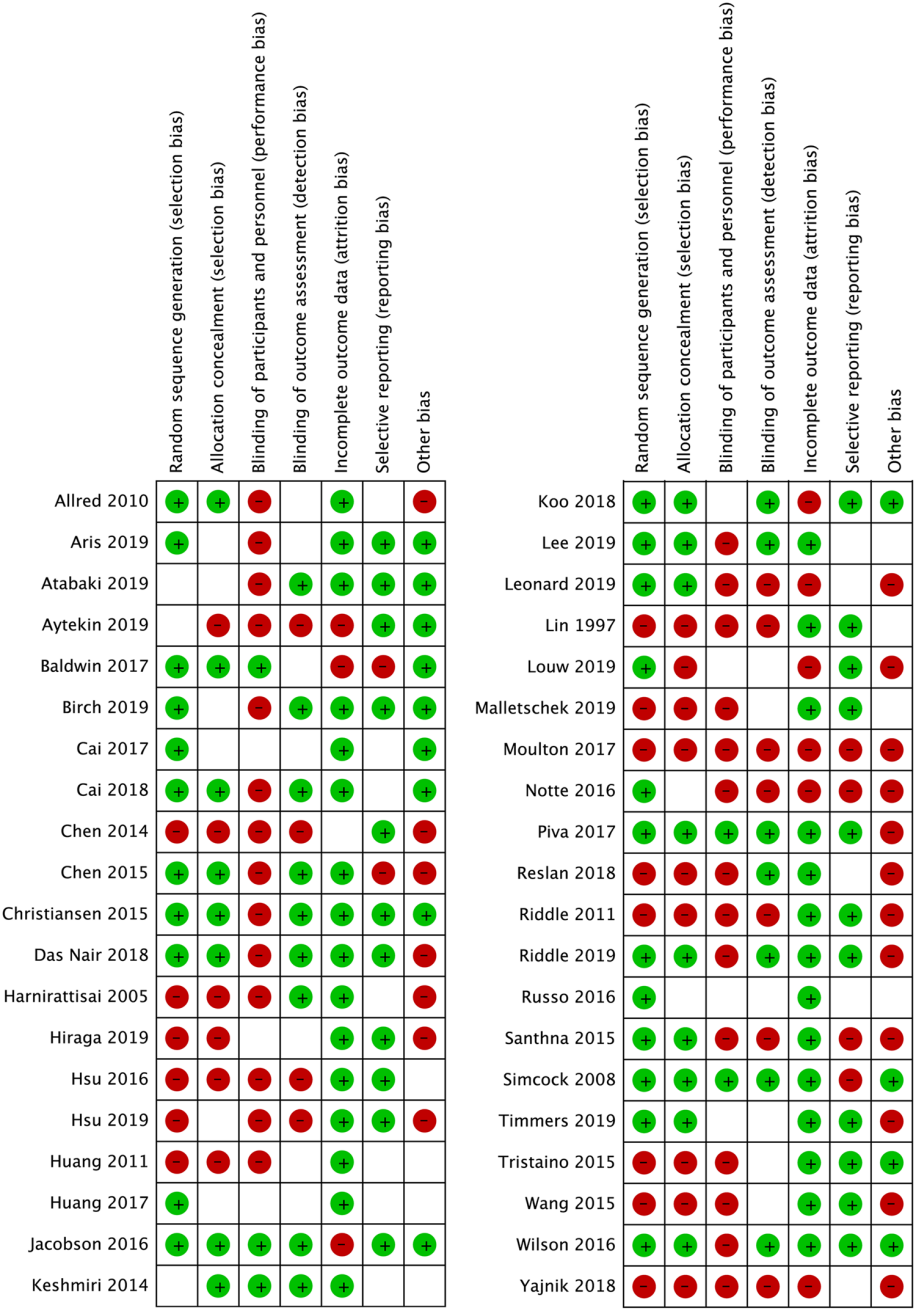
Risk of bias summary. Authors' judgements about each risk of bias item for each included study. Green: low risk of bias. Red: high risk of bias. No fill: unclear risk of bias

**Fig. 3:**
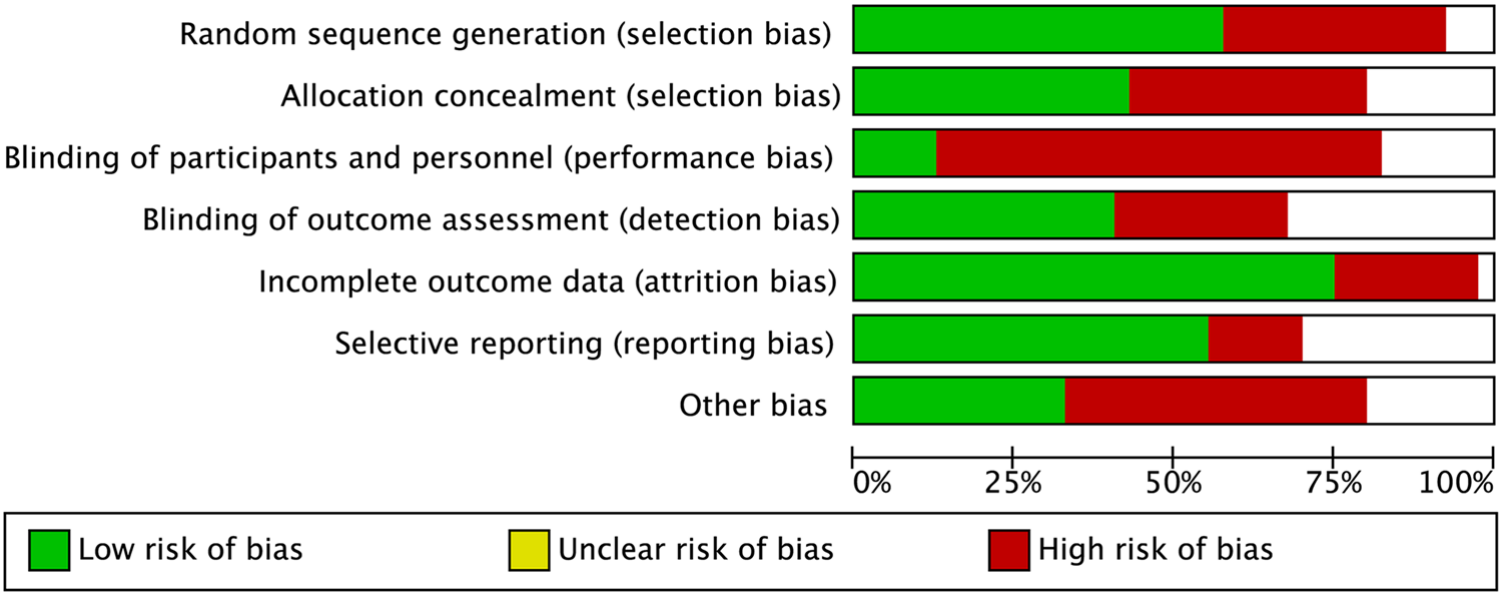
Risk of bias graph. Authors' judgements about each risk of bias item presented as percentages across all included studies. Green: low risk of bias. Red: high risk of bias. No fill: unclear risk of bias

Figure 2 shows our risk of bias assessment of the included studies. Figure 3 represents our judgement about each risk of bias item presented as percentages across all studies. The most prevalent shortcomings regarding the risk of bias were inadequate blinding participants and/or personnel during the study (performance bias) and “other types of bias”. Bias due to inadequate generation of a randomisation sequence or inadequate allocation concealment prior to assignment (selection bias) also caused high scores on the risk of bias (Fig. 3).

The overall level of evidence of the studies using the GRADE approach was qualified as low for pain and for function and as moderate for QoL. Serious uncertainty in the assessment of the risk of bias, inconsistency, and indirectness were the main reasons for downgrading the overall level of evidence (Table 5).

**Table 5:** The overall level of evidence using the GRADE approach

Certainty assessment												No of patients			Certainty
No of studies	Study design	Risk of bias			Inconsistency		Indirectness	Imprecision	Other considerations			ITPD		No ITPD	
Pain (follow up: range 60 min to 6 months; assessed with: Various outcome measures)															
34	19 randomised trials and 15 remaining*			Serious	Serious	Serious		Not serious		all plausible residual confounding would suggest spurious effect, while no effect was observed		1618	996		⨁⨁◯◯ low
Function (follow up: range 2 days to 2 years; assessed with: Various outcome measures)															
29	16 randomised trials and 13 remaining**		Serious		Serious	Serious		Not serious			all plausible residual confounding would suggest spurious effect, while no effect was observed	1580	1003		⨁⨁◯◯ low
QoL (follow up: range 24 weeks to 3 months; assessed with: Various outcome measures)															
2	1 randomised trial and one non-randomised trial		Serious		Serious	Not serious		Not serious			all plausible residual confounding would suggest spurious effect, while no effect was observed	151	137		⨁⨁⨁◯ moderate

*GRADE* grading of recommendation, assessment, development, and evaluation, *№* number, *ITPD* intervention targeting psychological distress, *QoL* quality of life

^*^8 prospective cohort studies, 6 quasi-experimental studies, 1 retrospective cohort study

^**^6 prospective cohort studies, 6 quasi-experimental studies, 1 retrospective cohort study

## Discussion

In this systematic review, we give an overview of studies that assessed the effect of perioperative interventions targeting psychological distress on pain, function, and QoL applied to patients undergoing TKA for primary OA of the knee. Perioperative music [33, 36, 38–40], education [41, 49, 51–53, 55], cognitive behavioural therapy [57, 58], pain coping skills training [62], guided imagery [61], perioperative Reiki therapy [66, 70], occupational therapy in combination with self-monitoring using a diary [68], and biofeedback-assisted progressive muscle relaxing training [71] seem to improve postoperative pain or to decline opioid prescriptions after TKA. For function, pain coping skills training [62], audiorecording guided imagery scripts [61], video promoting self-confidence and psychological support [64], music [35, 36], occupational therapy in combination with self-monitoring using a diary [68], various types of education [41, 43, 45, 47, 51–53], weight-bearing biofeedback training [67], psychological therapies (behavioural change intervention [60] and cognitive behavioural therapy [57–59]) seem to significantly improve at least one postoperative functional outcome measure. Day-to-day education after TKA using an app might improve postoperative QoL.

This is a methodologically well-conducted systematic review for which a professional medical librarian (CdH) has developed the search strategy to conduct a comprehensive search in several databases to identify eligible studies. Two authors (JS & GO) performed the screening, data extraction, risk of bias assessment, and overall level of evidence grading independently. We have created a complete overview of all studies by minimizing our exclusion criteria regarding study design, minimum follow-up, and language. Studies without significant results on the effect of an intervention are often refused for publication. Due to the heterogeneity of the outcome measures of the included studies, it was not possible to conduct a funnel plot to assess this type of bias (publication bias) in our systematic review. However, we included multiple studies [32–34, 38, 39, 42, 46, 55, 56, 68] with small sample sizes (smaller than 30 patients) with no significant results on both outcome measures pain and function. Therefore we assume the risk of publication bias to be low.

Unfortunately, drawing meaningful conclusions from the included studies was hampered. First of all, there was a substantial heterogeneity with respect to study design, analysis, domain, interventions, and outcome measures, which precluded pooling for a meta-analysis. Second, according to the GRADE approach, we have graded the quality of evidence as low for outcome measures pain and function. Therefore, the true effect of the interventions targeting psychological distress on postoperative pain and function may be different from our estimate of the effect.

The previous systematic reviews of Szeverenyi et al. [26] and Tong et al. [27] concluded that psychological interventions seem to reduce postoperative side effects and anxiety and to improve recovery and mental components of quality of life after orthopaedic surgeries. However, Szeverenyi et al. [Sweverenyi] did not clarify the type of orthopaedic procedures (only joint replacement or no joint replacement) and Tong et al. [27] included several orthopaedic procedures (THA, TKA, and spinal procedures) of which only two studies [61, 63] represented separated data of patients undergoing TKA. The findings of our review do not support the earlier systematic review of Bay et al. [25], in which most interventions explored by the included studies were found to be ineffective on patient-reported outcome after THA and TKA. Only three studies with patients receiving TKA were included by Bay et al. [25]. Compared to that review, we included fifteen additional RCTs [33, 34, 37, 38, 41, 44, 45, 49, 53, 54, 56–58, 58, 63]. Second, due to the current lack of RCTs on one specific type of intervention focused on psychological distress (for example only pain coping skills training) applied to patients undergoing TKA, we have decided to also include a wider range of study designs to create a complete overview of the perioperative interventions focused on psychological distress that have been used to decrease pain and improve function and/or QoL after surgery. Besides, ten studies [32, 34, 37, 39, 48, 54, 55, 66, 70, 71] in our systematic review evaluated the degree of postoperative pain not only by measuring pain scores, but also by assessing postoperative prescription of opioids or other types of pain medication. Investigating alternative nonpharmacologic methods to reduce postoperative pain and opioid use may help prevent further expansion of opioid misuse and addiction, which is currently a rapidly evolving public health crisis [7].

To the best of our knowledge, except for the mentioned systematic reviews [25, 26], no other systematic reviews or meta-analysis with comparable objectives have been published. Therefore, this is the first systematic review with wide search and inclusion criteria focused on TKA patients investigating the effect of interventions focused on psychological distress on patient-reported outcome measures pain, function, and QoL after surgery. Unfortunately, our review also highlighted the limitations of current literature on this subject. To avoid heterogeneity of outcome measures between studies, we would discourage the use of different questionnaires to assess patient-reported outcome measures (PROMs) in orthopaedic research. The reliability and reproducibility of the EuroQOL Five-Dimensional Questionnaire (EQ-5D) and the responsiveness of the Patient-Reported Outcomes Measurement Information System (PROMIS) Global Health survey have been well validated for patients undergoing TKA [72]. We would, therefore, recommend the use of the EQ-5D and PROMIS to allow tracking and evaluation of the effectiveness of perioperative interventions for psychological distress in conjunction with TKA in the following studies [72].

## Conclusions

The studies included in our systematic review show the positive effect of multiple perioperative interventions targeting psychological distress for patients receiving TKA to improve postoperative pain (or to decline prescriptions of opioids), function, and QoL. RCTs with strict methodological safeguards (such as long-term follow-up, large number of patients participating in the study, low risk of bias) prospectively comparing outcome for patients with and without perioperative support are still needed to determine if perioperative interventions targeting psychological distress should be used in conjunction with primary TKA for OA of the knee. These studies should also assess which type of intervention will be most effective in improving patient-reported outcome measures and declining opioid prescriptions in the future.

## Electronic supplementary material

Below is the link to the electronic supplementary material.

Supplementary file1 (DOCX 117 kb)
