# Supplementary material for: The influence of perioperative interventions targeting psychological distress on clinical outcome after total knee arthroplasty
Source: Rheumatol Int. 2020 Jul 29;40(12):1961–86. doi: 10.1007/s00296-020-04644-y (PMC7591436; doi:10.1007/s00296-020-04644-y)
Supplement: Supplementary file 1 — Supplementary file1 (DOCX 117 kb) [file 296_2020_4644_MOESM1_ESM.docx]

### PubMed

| **No.** | **Query** |
| --- | --- |
| #3 | #1 AND #2 |
| #2 | (("Self Care"[Mesh] OR "Adaptation, Psychological"[Mesh] OR "Emotions"[Mesh] OR "Stress, Psychological"[Mesh] OR "Emotional Adjustment"[Mesh] OR "Catastrophization"[Mesh] OR "Self Efficacy"[Mesh] OR anxiety[tiab] OR anxious*[tiab] OR behavio*[tiab] OR catastrophi*[tiab] OR CBT[tiab] OR cognit*[tiab] OR coping[tiab] OR counseling[tiab] OR Dialectical behavio*[tiab] OR emotion*[tiab] OR e-therapy[tiab] OR etherapy[tiab] OR hypno*[tiab] OR MBCT[tiab] OR MBSR[tiab] OR mhealth[tiab] OR mobile health[tiab] OR meditat*[tiab] OR mindfulness[tiab] OR peri-operative educat*[tiab] OR perioperative educat*[tiab] OR preoperative educat*[tiab] OR pre-operative educat*[tiab] OR psychotherap*[tiab] OR psychological*[tiab] OR psychosoc*[tiab] OR relaxation[tiab] OR self-efficacy[tiab] OR selfefficacy[tiab]) AND ("Therapeutics"[Mesh] OR "therapy" [Subheading] OR "education" [Subheading] OR intervention*[tiab] OR program*[tiab] OR therapy[tiab] OR training[tiab] OR treatment*[tiab] OR support[tiab])) OR "Psychotherapy"[Mesh:NoExp] OR "Behavior Therapy"[Mesh:NoExp] OR "Cognitive Behavioral Therapy"[Mesh] OR "Psychotherapy, Group"[Mesh:NoExp] OR "Hypnosis"[Mesh] OR "Telemedicine"[Mesh] OR "Relaxation"[Mesh] OR "Relaxation Therapy"[Mesh] OR "Mind-Body Therapies"[Mesh:NoExp] OR "Counseling"[Mesh] OR "Health Education"[Mesh] |
| #1 | "Arthroplasty, Replacement, Knee"[Mesh] OR "Knee Prosthesis"[Mesh] OR TKA[tiab] OR TKR[tiab] OR (("Knee Joint"[Mesh] OR "Knee"[Mesh] OR "Joints"[Mesh] OR knee[tiab] OR knees[tiab] OR joint[tiab]) AND ("Arthroplasty, Replacement"[Mesh] OR replacement*[tiab] OR arthroplast*[tiab])) |

### Embase.com

| **No.** | **Query** |
| --- | --- |
| #7 | #1 AND #6 |
| #6 | #4 OR #5 |
| #5 | 'psychotherapy'/de OR 'behavior therapy'/exp OR 'behavior modification'/exp OR 'cognitive therapy'/exp OR 'group therapy'/exp OR 'hypnosis'/exp OR 'telemedicine'/exp OR 'relaxation training'/exp OR 'counseling'/exp OR 'health education'/exp |
| #4 | #2 AND #3 |
| #3 | 'therapy'/exp OR intervention*:ab,ti OR program*:ab,ti OR therapy:ab,ti OR training:ab,ti OR treatment*:ab,ti OR support:ab,ti |
| #2 | 'self care'/exp OR 'adaptive behavior'/exp OR 'emotion'/exp OR 'mental stress'/exp OR 'psychological adjustment'/exp OR 'catastrophizing'/exp OR 'self concept'/exp OR anxiety:ab,ti OR anxious*:ab,ti OR behavio*:ab,ti OR catastrophi*:ab,ti OR cbt:ab,ti OR cognit*:ab,ti OR coping:ab,ti OR counseling:ab,ti OR ((dialectical NEXT/1 behavio*):ab,ti) OR emotion*:ab,ti OR 'e therapy':ab,ti OR etherapy:ab,ti OR hypno*:ab,ti OR mbct:ab,ti OR mbsr:ab,ti OR mhealth:ab,ti OR 'mobile health':ab,ti OR meditat*:ab,ti OR mindfulness:ab,ti OR (('peri operative' NEXT/1 educat*):ab,ti) OR ((perioperative NEXT/1 educat*):ab,ti) OR ((preoperative NEXT/1 educat*):ab,ti) OR (('pre operative' NEXT/1 educat*):ab,ti) OR psychotherap*:ab,ti OR psychological*:ab,ti OR psychosoc*:ab,ti OR relaxation:ab,ti OR 'self-efficacy':ab,ti OR selfefficacy:ab,ti |
| #1 | 'knee arthroplasty'/exp OR 'knee prosthesis'/exp OR tka:ab,ti OR tkr:ab,ti OR (('joint'/exp OR knee:ab,ti OR knees:ab,ti OR joint:ab,ti) AND ('arthroplasty'/exp OR replacement*:ab,ti OR arthroplast*:ab,ti)) |

### PsycInfo via Ovid

| **No.** | **Query** |
| --- | --- |
| #1 | ((knee/ OR "joints (anatomy)"/) AND (surgery/ OR surgery.ti,ab,id.)) OR (((knee OR knees OR joint) ADJ3 (surgery OR replacement* OR transplant* OR repair* OR operation OR reduction OR orthop?edic* OR arthroplast* OR arthroscop*)) OR TKA OR TKR).ti,ab,id. |

### Cochrane Library

| **No.** | **Query** |
| --- | --- |
| #1 | MeSH descriptor: [Arthroplasty, Replacement, Knee] explode all trees |
| #2 | MeSH descriptor: [Knee Prosthesis] explode all trees |
| #3 | (TKA or TKR):ti,ab,kw |
| #4 | #1 OR #2 OR #3 |
| #5 | MeSH descriptor: [Knee Joint] explode all trees |
| #6 | MeSH descriptor: [Knee] explode all trees |
| #7 | MeSH descriptor: [Joints] explode all trees |
| #8 | (knee or knees or joint):ti,ab,kw |
| #9 | #5 or #6 or #7 or #8 |
| #10 | MeSH descriptor: [Arthroplasty, Replacement] explode all trees |
| #11 | (replacement* or arthroplast*):ti,ab,kw |
| #12 | #10 or #11 |
| #13 | #9 and #12 |
| #14 | #4 or #13 |
| #15 | MeSH descriptor: [Self Care] explode all trees |
| #16 | MeSH descriptor: [Adaptation, Psychological] explode all trees |
| #17 | MeSH descriptor: [Emotions] explode all trees |
| #18 | MeSH descriptor: [Stress, Psychological] explode all trees |
| #19 | MeSH descriptor: [Emotional Adjustment] explode all trees |
| #20 | MeSH descriptor: [Catastrophization] explode all trees |
| #21 | MeSH descriptor: [Self Efficacy] explode all trees |
| #22 | (((anxiety or anxious* or behavio* or catastrophi* or CBT or cognit* or coping or counseling or (Dialectical next behavio*) or emotion* or “e-therapy” or etherapy or hypno* or MBCT or MBSR or mhealth or “mobile health” or meditat* or mindfulness or (peri-operative next educat*) or (perioperative next educat*) or (preoperative next educat*) or (pre-operative next educat*) or psychotherap* or psychological* or psychosoc* or relaxation or self-efficacy or selfefficacy))):ti,ab,kw |
| #23 | {or #15-#22} |
| #24 | MeSH descriptor: [Therapeutics] explode all trees |
| #25 | MeSH descriptor: [] explode all trees and with qualifier(s): [education - ED, therapy - TH] |
| #26 | (intervention* or program* or therapy or training or treatment* or support):ti,ab,kw |
| #27 | {or #24-#26} |
| #28 | #23 and #27 |
| #29 | MeSH descriptor: [Psychotherapy] explode all trees |
| #30 | MeSH descriptor: [Behavior Therapy] explode all trees |
| #31 | MeSH descriptor: [Cognitive Behavioral Therapy] explode all trees |
| #32 | MeSH descriptor: [Psychotherapy, Group] explode all trees |
| #33 | MeSH descriptor: [Hypnosis] explode all trees |
| #34 | MeSH descriptor: [Telemedicine] explode all trees |
| #35 | MeSH descriptor: [Relaxation] explode all trees |
| #36 | MeSH descriptor: [Relaxation Therapy] explode all trees |
| #37 | MeSH descriptor: [Mind-Body Therapies] explode all trees |
| #38 | MeSH descriptor: [Counseling] explode all trees |
| #39 | MeSH descriptor: [Health Education] explode all trees |
| #40 | {or #28-#39} |
| #41 | #14 AND #40 |
| #42 | #14 AND #40 in Cochrane Reviews |
| #43 | #14 AND #40 in Trials |

### Web of Science

| **No.** | **Query** |
| --- | --- |
| # 5 | #4  AND  #1  Indexes=SCI-EXPANDED, SSCI, A&HCI, ESCI Timespan=All years |
| # 4 | #3  AND  #2  Indexes=SCI-EXPANDED, SSCI, A&HCI, ESCI Timespan=All years |
| # 3 | **TOPIC:**  (intervention* OR program* OR therapy OR training OR treatment* OR support)  Indexes=SCI-EXPANDED, SSCI, A&HCI, ESCI Timespan=All years |
| # 2 | **TOPIC:**  (anxiety OR anxious* OR behavio* OR catastrophi* OR cbt OR cognit* OR coping OR counseling OR dialectical-behavio* OR emotion* OR 'e therapy' OR etherapy OR hypno* OR mbct OR mbsr OR mhealth OR mobile-health OR meditat* OR mindfulness OR peri-operative-educat* OR perioperative-educat* OR preoperative-educat* OR pre-operative-educat* OR psychotherap* OR psychological* OR psychosoc* OR relaxation OR self-efficacy OR selfefficacy)  Indexes=SCI-EXPANDED, SSCI, A&HCI, ESCI Timespan=All years |
| # 1 | **TOPIC:**  (TKA OR TKR OR ((knee OR knees OR joint)  AND  (replacement* OR arthroplast*) ))  Indexes=SCI-EXPANDED, SSCI, A&HCI, ESCI Timespan=All years |

### Scopus

| **No.** | **Query** |
| --- | --- |
| 5 | ( TITLE-ABS-KEY ( tka  OR  tkr  OR  ( ( knee  OR  knees  OR  joint )  AND  ( replacement*  OR  arthroplast* ) ) ) )  AND  ( ( ( TITLE-ABS-KEY ( anxiety  OR  anxious*  OR  behavio*  OR  catastrophi*  OR  cbt  OR  cognit*  OR  coping  OR  counseling  OR  "dialectical behavio*"  OR  emotion*  OR  "e therapy"  OR  etherapy  OR  hypno*  OR  mbct  OR  mbsr  OR  mhealth  OR  "mobile health"  OR  meditat*  OR  mindfulness )  OR  TITLE-ABS-KEY ( "peri-operative educat*"  OR  "perioperative educat*"  OR  "preoperative educat*"  OR  "pre operative educat*"  OR  psychotherap*  OR  psychological*  OR  psychosoc*  OR  relaxation  OR  "self efficacy"  OR  selfefficacy ) ) )  AND  ( TITLE-ABS-KEY ( intervention*  OR  program*  OR  therapy  OR  training  OR  treatment*  OR  support ) ) )  ...View More |
| 4 | ( ( TITLE-ABS-KEY ( anxiety  OR  anxious*  OR  behavio*  OR  catastrophi*  OR  cbt  OR  cognit*  OR  coping  OR  counseling  OR  "dialectical behavio*"  OR  emotion*  OR  "e therapy"  OR  etherapy  OR  hypno*  OR  mbct  OR  mbsr  OR  mhealth  OR  "mobile health"  OR  meditat*  OR  mindfulness )  OR  TITLE-ABS-KEY ( "peri-operative educat*"  OR  "perioperative educat*"  OR  "preoperative educat*"  OR  "pre operative educat*"  OR  psychotherap*  OR  psychological*  OR  psychosoc*  OR  relaxation  OR  "self efficacy"  OR  selfefficacy ) ) )  AND  ( TITLE-ABS-KEY ( intervention*  OR  program*  OR  therapy  OR  training  OR  treatment*  OR  support ) )  ...View More |
| 3 | TITLE-ABS-KEY ( intervention*  OR  program*  OR  therapy  OR  training  OR  treatment*  OR  support ) |
| 2 | ( TITLE-ABS-KEY ( anxiety  OR  anxious*  OR  behavio*  OR  catastrophi*  OR  cbt  OR  cognit*  OR  coping  OR  counseling  OR  "dialectical behavio*"  OR  emotion*  OR  "e therapy"  OR  etherapy  OR  hypno*  OR  mbct  OR  mbsr  OR  mhealth  OR  "mobile health"  OR  meditat*  OR  mindfulness )  OR  TITLE-ABS-KEY ( "peri-operative educat*"  OR  "perioperative educat*"  OR  "preoperative educat*"  OR  "pre operative educat*"  OR  psychotherap*  OR  psychological*  OR  psychosoc*  OR  relaxation  OR  "self efficacy"  OR  selfefficacy ) )  ...View More |
| 1 | TITLE-ABS-KEY ( tka  OR  tkr  OR  ( ( knee  OR  knees  OR  joint )  AND  ( replacement*  OR  arthroplast* ) ) ) |
